# Coordination of the health policy dialogue process in Guinea: pre- and post-Ebola

**DOI:** 10.1186/s12913-016-1457-8

**Published:** 2016-07-18

**Authors:** Nadege Ade, Adzodo Réne, Mara Khalifa, Kevin Ousman Babila, Martin Ekeke Monono, Elongo Tarcisse, Juliet Nabyonga-Orem

**Affiliations:** 1 Heath Systems Analyst and Facilitator, Community of Practice-Health Systems Planning & Budgeting, Nice, 06200 France; 2 Health Systems and Services, World Health Organization Guinea Country Office, PO Box Boîte postale 817, Conakry, Guinea; 3 Health Systems and Services Cluster, World Health Organization Regional Office for Africa, B.P. 06, Brazzaville, Congo

**Keywords:** Health policy dialogue, Coordination, Pre and post Ebola

## Abstract

**Background:**

Policy dialogue can be defined as an iterative process that involves a broad range of stakeholders discussing a particular issue with a concrete purpose in mind. Policy dialogue in health is increasingly being recognised by health stakeholders in developing countries, as an important process or mechanism for improving collaboration and harmonization in health and for developing comprehensive and evidence-based health sector strategies and plans. It is with this perspective in mind that Guinea, in 2013, started a policy dialogue process, engaging a plethora of actors to revise the country’s national health policy and develop a new national health development plan (2015–2024). This study examines the coordination of the policy dialogue process in developing these key strategic governance documents of the Guinean health sector from the actors’ perspective.

**Methods:**

A qualitative case study approach was undertaken, comprising of interviews with key stakeholders who participated in the policy dialogue process. A review of the literature informed the development of a conceptual framework and the data collection survey questionnaire. The results were analysed both inductively and deductively.

**Results:**

A total of 22 out of 32 individuals were interviewed. The results suggest both areas of strengths and weaknesses in the coordination of the policy dialogue process in Guinea. The aspects of good coordination observed were the iterative nature of the dialogue and the availability of neutral and well-experienced facilitators. Weak coordination was perceived through the unavailability of supporting documentation, time and financial constraints experienced during the dialogue process. The onset of the Ebola epidemic in Guinea impacted on coordination dynamics by causing a slowdown of its activities and then its virtual halt.

**Conclusions:**

The findings herein highlight the need for policy dialogue coordination structures to have the necessary administrative and institutional support to facilitate their effective functioning. The findings also point to the need for further research on the practical and operational aspects of national dialogue coordination structures to determine how to best strengthen their capacities.

## Background

Policy dialogue is an approach that has recently captured interest and attention in public health policy, most probably due to its perceived capacity to address complex issues and facilitate evidence-based policy-making and decision making [1]. The term policy dialogue has different meanings to different people and in different contexts. It has been defined as an “event” where dialogue takes place around a policy question using evidence. It may also be perceived as a “process” of deliberative dialogues around a policy brief [2]. Policy dialogue has seen a fairly good level of implementation and documentation in developed countries [3–6] and it is increasingly being recognised by national stakeholders and development partners in low and middle income countries, (including countries of sub-Saharan Africa), as an important process or mechanism to engage a plethora of actors within and beyond the health sector on key issues around adequate health sector development and effective health systems strengthening.

**Fig. 1:**
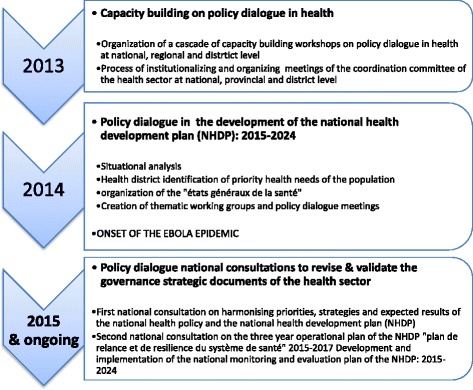
Policy dialogue roadmap of activities and timelines in Guinea

The republic of Guinea has been strengthening its health policy dialogue process which effectively began in 2013, under a programme of the European Union-and the World Health Organization (WHO), with the main objectives of revising the national health policy, and developing a new national health development plan. With the ending of the (2003–2012) plan, a new one had to be formulated to guide the development of the health sector. Organising policy dialogues involving a multitude of stakeholders working in the health sector was therefore seen as key in formulating a robust, comprehensive and evidence-based national health development plan. In preparation for implementing the policy dialogue process, a coordination committee (Comité de coordination du secteur de la santé, CCSS) consisting of a broad range of actors within the health sector, was created in 2012 under the authority of the country’s prime minister. This committee was to coordinate the policy dialogue process through a roadmap of activities which it had developed. Furthermore, the committee was to be supported by a technical secretariat as well as health sector technical working groups in implementing the roadmap of activities. Figure 1 portrays these activities.

The policy dialogue in Guinea was implemented at various levels of the health system and a multitude of actors were involved at both national and subnational levels. These actors included health officials of the Ministry of Health (MoH) at national and sub-national levels, civil society organizations, representatives from development partners, as well as actors from other sectors, such as the Ministry of Environment. In March 2014, a year into the implementation of the policy dialogue process, the Ebola epidemic in Guinea was officially declared [7]. Prior to the outbreak, certain key activities of the dialogue process had already been undertaken such as the organization of capacity building workshops on policy dialogue at central and sub national (regional) levels and the creation of the health thematic working groups. A preliminary situational analysis of the health sector had also been performed, an activity which laid the groundwork for the “états généraux de la santé”, a meeting of close to 150 health professionals from various cadre of health sector, under the leadership of the President of the Republic. This was to decipher the underlying causes of the weak performance of the health system, identify the priority health needs of the population and to inform the revision of the country’s national health policy, and formulate the new national health development plan- 2015–2024. However, the process of giving an institutional backbone to the coordination committee of the health sector at the national, regional and district levels had not yet been completed. It is within this context of a still evolving health policy dialogue process that the Ebola epidemic occurred.

This study had as main objective, to assess the perceptions of the Guinean stakeholders who participated in this health policy dialogue process. Because the Ebola epidemic occurred while the policy dialogue process was being implemented, the paper also presents actors’ perceptions of how the coordination process was affected (or not) by the Ebola epidemic. This paper does not intend to compare coordination of the policy dialogue process prior to- and post-Ebola, neither does it seek to assess the results or outcomes of the dialogue process. Rather, it provides an in-depth look into actors’ perceptions of the process they were involved in, and how the onset of the Ebola epidemic may (or may not) have influenced the coordination of this policy dialogue process. To our knowledge, this study is the first of its kind to directly assess policy dialogue coordination in the context of low- and middle-income countries. The findings of the study will make an important contribution to the understanding of policy dialogue and its key features. It will also provide key insights to the Guinean government and other countries or institutions, interested in strengthening policy dialogue, as a mechanism for engaging all stakeholders in health sector development.

## Methods

### Interview guide development

**Fig. 2:**
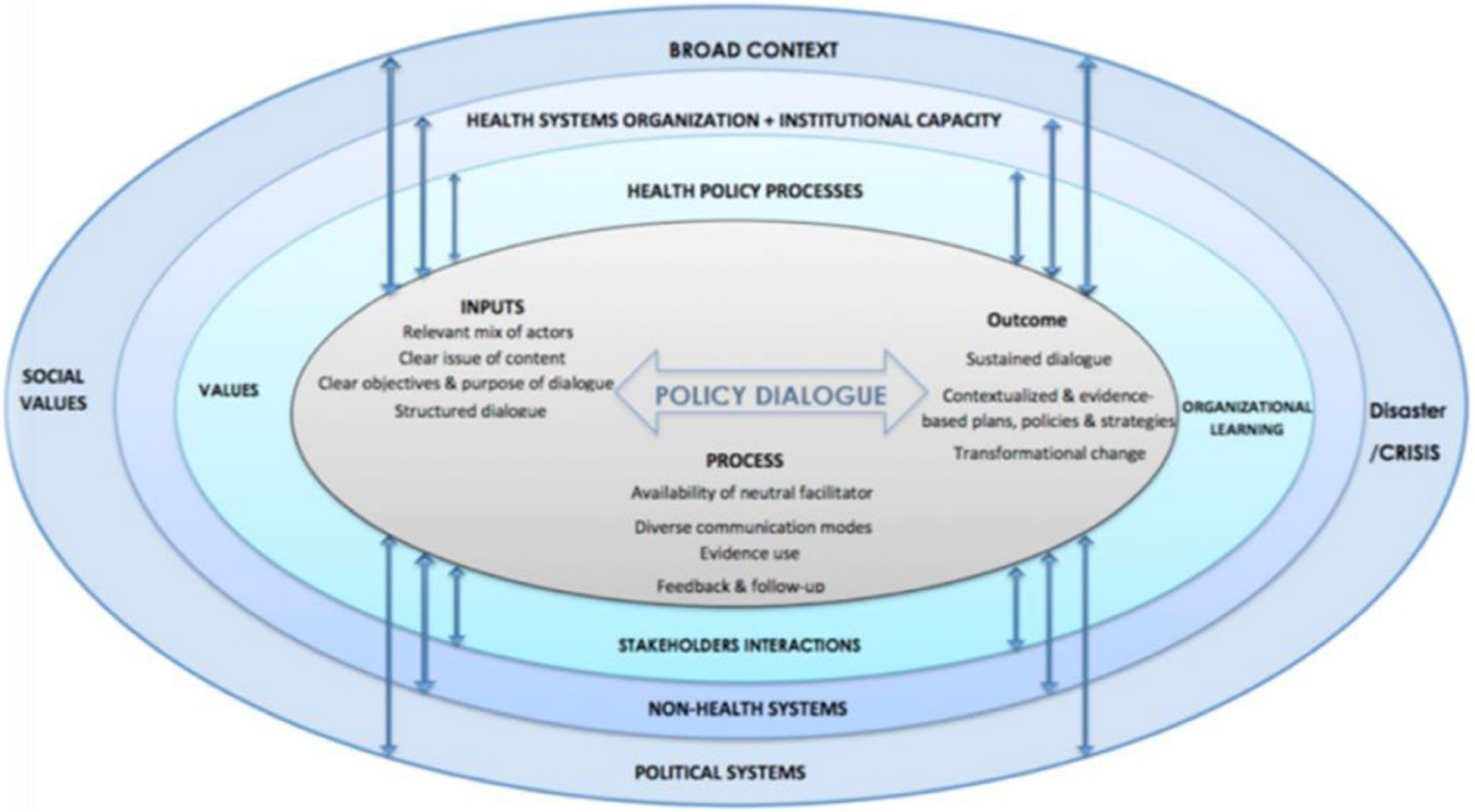
Conceptual framework for health policy dialogue

An initial document review informed the development of an interview guide. A non-exhaustive review of the published literature on policy dialogue (searching Google, Google Scholar, PubMed and HINARI using search terms: ‘policy dialogue’, ‘deliberative dialogue’ and ‘deliberative process’), and review of national-level strategic planning documents (including the national development health plan, and annual reports) was conducted. Based on this we developed our conceptual framework for the study (Fig. 2 below) as shown in Nabyonga-Orem et al, this issue [8]. The framework proposes key elements important for policy dialogue processes: inputs necessary for policy dialogue, processes of implementing the dialogue, and outcomes of the dialogue. This article focuses on actor perceptions of the coordination of the health policy dialogue in terms of its "inputs" and the "process".

From the document review and conceptual framework, an interview guide was developed. The first part of the interview sought to elicit information on the conceptualisation and implementation design of the policy dialogue process (i.e., respondent understandings of policy dialogue, respondent perspectives on improved alignment among stakeholders, improved collaboration among stakeholders, and values and attitudes propagated by the policy dialogue). The second section contained questions on the policy dialogue process at the onset of the Ebola epidemic (i.e., governance and oversight issues addressed by policy dialogue prior to Ebola, changes to the nature of the policy dialogue after the onset of Ebola, differences in national and subnational responses). The interview questions were validated among WHO officers in the Guinea WHO office, and stakeholders who had participated in the process but were not going to be interviewed, to ensure contextual accuracy and clarity. A purposive sampling approach was used to select participants on the basis of their active involvement in the policy dialogue process. Key stakeholders were identified by WHO country officers and the Guinean MoH as lead coordinators of the policy dialogue process. Additional stakeholders were identified through a snowballing until descriptive saturation was achieved [9].

### Study participants

Thirty two respondents were identified at national and subnational (i.e., regional) levels and a total of 22 interviews were conducted. The number and profile of the respondents are shown in Table 1.

**Table 1:** Table showing number and profile of respondents

Institution	Number
National-level	
Ministry of Health	10
Civil society	2
Development partners	6
Ministry of Environment	1
Subnational level	
Regional level MOH	2
Civil society	1
Total number of respondents	22

Three local researchers conducted the interviews which lasted averagely 45 to 60 min. Interviews were conducted from August and September 2015 and were conducted mostly at the respondents’ workplace. All the interviewees agreed to electronic recording, but for the five key informants who could not be physically available, the interview responses were typed out and sent to the researchers by email. Interviews were later transcribed verbatim and analysed using inductive content analysis, and kept confidential on a security-protected computer.

## Results

### Coordination of the policy dialogue process

#### Availability of key policy dialogue inputs

The coordination of the policy dialogue process in Guinea was generally seen by the majority of the respondents as well conducted owing to the participation of a relevant mix of actors in the process, and the dialogue process having clear objectives and a defined purpose. Most respondents repeatedly stated that the process was participatory; as they had felt free to speak their mind and share their points of view and that they were heard by all those present.

*“[It was]… an inclusive dialogue because all the stakeholders (civil society, public sector, private sector and para-public sector, NGOs, bi-lateral and multi-lateral partners, health users) were well represented, so the group was multi-sectoral and multidisciplinary and a diversity of themes was addressed” (respondent I)*

*“The policy dialogue was well conducted. When they made us understand that the work could not be done without the involvement of civil society organisations, it made me realise the important role and contribution that we, civil society organisations have in the development of these documents”.(respondent II)*

Similarly, when asked about the objectives and goals of the policy dialogue process, almost all the participants believed that the issue of contention and the objectives of the dialogue were clear and explicit in terms of the goal of developing governance tools and reference documents for the health sector. *“The terms of reference clarified the objectives and expected outcomes of the health policy dialogue process”. (respondent III)*

Despite the fact that the policy dialogue process was generally perceived as very participatory, there were different perspectives on the level of inclusiveness. For example, the process of engaging relevant stakeholders could have been more inclusive if it had adopted a bottom-top approach, where actors from the district-level determined the priorities of the health sector, with these discussed and analysed later at the national level. The process actually adopted a top-down approach, where health actors at the national level developed a draft of the health sector priority plan and shared it with the actors at the prefectural and regional levels for comments and amendments.

“*In my point of view, it should have started with communities at the level of health centres, going up to the prefectural, regional and national level. Instead of following this schema, the approach was rather top-down. We first developed a national plan which was submitted to structures at regional level for comments and amendments. In this respect, the dialogue was not very participative” (respondent V)*

When the respondents were asked which structure or institution coordinated the dialogue process, they had varied responses, demonstrating a clear lack of awareness of which institution had the coordination role (Table 2). Respondents answered variably that WHO, Ministry of Health (MOH), both WHO and MOH, or some other organisation was responsible for coordination. One respondent indicated they were unaware who coordinated the policy dialogues.

**Table 2:** Table showing key informants’ responses to the question “which institution coordinated the policy dialogue process”?

Institution	Number of respondents
World Health Organization (WHO)	6
Ministry of Health (MoH)	9
MoH & WHO	4
MoH, WHO & EU	2
Coordinating Committee of the Health sector (CCSS)	1
An agency of the United Nation or the European Union	1
World Bank	1

Of concern is the fact that the entity mandated to coordinate the health policy dialogue, the CCSS, was least known to the majority of respondents. Of interest was the finding that actors’ were more likely to name their affiliated institution as the coordinator of the dialogue process. One respondent said:

*“It’s the WHO that initiated and coordinated the health policy dialogue process. Its role was to sensitise a group of actors at the level of the ministry of health, certain partners and institutions of the republic in order to facilitate the policy dialogue meeting”* (respondent VI)

Yet another said:

*“It’s the ministry of health, the WHO and the EU. The role played by these institutions is one of catalysis and orientation” (respondent VII).*

And still:

*“It’s the coordination committee of the health sector at national level. It played a coordinative role to facilitate information sharing”* (respondent VIII).

At subnational level, respondents reported that it was the Regional Directorates of Health (DRS) and the Prefectural Health Directorates (DPS) that coordinated the policy dialogue process.

### Implementation process of the policy dialogue

#### Policy dialogue coordination prior to the advent of Ebola

The implementation of the policy dialogue process consisted of a series of consultation meetings during which stakeholders discussed the health systems’ priorities that were reviewed during the “état généraux de la santé” with the aim of informing the preparation of the National Health Development Plan (NHDP:2015-2024). These consultations were preceded by meetings of the thematic working groups, which undertook preparatory work on the relevant issues to be discussed, and presented their findings for a general discussion among the actors present at the policy dialogue consultation meetings.

Almost all the respondents perceived that the policy dialogue process they had been engaged in was well-facilitated, notably, through the presence of well-experienced and neutral facilitators who guided the dialogue in an impartial way Two respondents noted:

“*The facilitators behaved in an impartial way and were neutral throughout the policy dialogue process which they structured in such a way as to enhance contribution from all stakeholders*” (respondent XIII)

*“The facilitators had the required level. They effectively conducted the debates in a neutral way” (respondent XIV)*

The respondents’ views were quite mixed as to whether the dialogue process was guided by or used relevant data and evidence. Some of them believed that the data available within the country were used to guide and inform the policy dialogue process, such as the results of the health sector analysis study and of the evaluation study on the previous national health development plan (2003–2012). One respondent stated that:

*“In the preparation of the working documents of the policy dialogue process, the results of analytical and evaluation studies and surveys were used….the analytical study of the health sector, the evaluation of the national health development plan and this enabled to adapt certain strategies or to define new ones” (respondent V)*.

Other respondents however believed that the process was not informed by adequate data and evidence, given that the situation analysis on the health sector had not been adequately performed and was not based on strong and reliable data:

*“The evidence based on national and international experiences were not sufficiently taken into consideration. We kept on using old patterns and schemas” (respondent XV)*

*“The dialogue was based more on the opinions and experiences of participants rather than on factual data or evidence” (respondent III)*

When asked whether they had experienced any difficulties during the implementation of the policy dialogue process, many respondents cited the unavailability of supporting documentation to guide the work of the thematic working groups and the dialogue process, as a key constraint.

“*We didn’t have the necessary documentation to do a better job. The group work sessions were fast with no supporting documentation*” (respondent VII) and another said: “*The constraint faced was the unavailability of a plan to guide the working groups*” (respondent VIII)

Another major constraint, which was cited by more than half of the participants pertained to financial insufficiencies and delays in accessing funds. Specific details on this issue included the lack of financial resources for the implementation of some key activities of the process and the lack of or delays in providing stipends and transport costs disbursements to the stakeholders who participated in the dialogue. These are highlighted in the following quotes:

“*Also, there were some delays with the finances, all the participants who were expecting some stipends left before the end of the workshops in certain places*” *(respondent XVI)*

*“There was a lack of financial resources for some activities” (respondent XVII)*

The time allocated to the dialogue was also seen by most participants as a key constraint. Many of them believed that the time frame for the stakeholders’ discussions and also for the overall activities of the policy dialogue process was insufficient.

“*The difficulty had to do with the time which was relatively short*” (respondent *XVIII)*

“*As regards to the difficulties experienced, it mostly had to do with the time which was relatively short and the financial resources which were not made available” (respondent XIX)*

These results suggest that while some technical aspects of the coordination of the policy dialogue process were performed well, such as the role played by the facilitators and – though to a lesser extent – the use of local data and evidence, some operational aspects of the coordination effort seemed challenging, notably the failure to allocate adequate time for the discussions and exchanges, the limited supporting documentation to guide the policy dialogue process, and the financial delays. As noted by one respondent,

*“In my opinion, it’s the coordination aspect that needs to be strengthened; it is the fundamental constraint that I observed”.* (respondent XI)

#### Policy dialogue coordination during the Ebola epidemic

The Ebola epidemic in Guinea arose during the implementation of the policy dialogue process. The expected outputs of the policy dialogue process, particularly the revision of the national health policy and the preparation of the new national health development plan had not yet been achieved. The 2015 national report, detailing the progress of the policy dialogue process, highlights that the process was stalled from the period when the Ebola epidemic was declared in March 2014 up until October 2014, when the WHO took definitive steps to revive the policy dialogue process and the works of the CCSS, through hiring an international health systems expert [10].

Most respondents’ views generally reflected this account of the impact of the Ebola epidemic on the coordination of the policy dialogue process.

“*The outbreak of the epidemic slowed down the implementation of certain activities such as the surveys aimed at enriching the data on situational analysis. It is only now that we are conducting these surveys. At a given moment, the committee in charge of piloting the dialogue could no longer meet because of Ebola: The prime minister’s office sponsoring all these structures interrupted its activities*” (respondent VII)

While the findings suggest that the coordination of the policy dialogue process did stall for a while owing to the Ebola epidemic, some respondents’ views seem to suggest that this occurred at a later stage of the epidemic not at its onset. The dialogue process seems to have continued during the early stages of the epidemic through a series of consultation meetings that put greater focus on Ebola than on the national health development plan. As respondents said:

“*The apparition of the Ebola epidemic led to better diagnosis and analysis of the health system. It opened the eyes of health decision makers on aspects such as community involvement which had not been adequately considered (during the policy dialogue)* (respondent XVII).

“*The early warning and response system and the consultation meetings among partners were discussed during the dialogue”.* (respondent V)

“*Every partner shared their experience. We for example we already had some community agents in our different sites. They asked us if it was not necessary to involve them in the village committees in charge of surveillance, to disseminate messages on Ebola prevention, which we did*” (respondent II)

These results suggest that at the onset of the epidemic, the CCSS changed gears from coordinating activities related to the development of the national health development plan to trying to coordinate the dialogue on issues pertaining the Ebola virus disease.

That situation did not last long, however, as a new multisectoral government structure, the Cellule nationale de coordination de la maladie à virus Ebola (CNRE) was created as the sole body with the mandate to coordinate all the activities related to Ebola response. This supra-national structure was directly accountable to the presidency, and all the other national bodies had to abide by the requirements relating to the coordinating role of this structure One respondent perceived this situation as “*replacing the coordination role of the CCSS with the CNRE*”, supporting the idea that in the early stages of the epidemic, the CCSS did put on hold it activities of coordinating the policy dialogue process on the national health development plan to try to coordinate the dialogue about the Ebola response.

*“An ad hoc coordinating structure was created by the government to deal with the national urgency. This national structure, directly reporting to the presidency, put on hold the functioning of the CCSS, which was no longer having meetings”. (respondent XX)*

Policy dialogue participants therefore perceived the rise of the Ebola epidemic as a factor that slowed down the policy dialogue process and then led to its halt when the coordination of the Ebola response was put solely into the hands of the CNRE. The CCSS stopped its activities and did not resume the coordination of the policy dialogue process on developing the national health development plan up until when an international expert was hired by the WHO to revive the dialogue process six months after the onset of the epidemic. Since then, and with the resumed work of the CCSS, two national policy dialogue consultations were organized and saw the final revision of the National Health Policy, and the finalisation of the national health development plan.

## Discussion

This study has key findings on actors’ perceptions of the coordination of the policy dialogue process in developing the governance and reference documents of the Guinean health sector. First, the policy dialogue was perceived as having been very well facilitated by experienced and neutral facilitators. The process was also seen as interactive, as the actors could freely talk and share their points of views. The intended purpose of the policy dialogue was clear to all participants. However the level of inclusiveness of the relevant actors in the dialogue was contested.

The respondents generally seemed to perceive the facilitation and participation aspects in a positive light, reflecting the notion that to them, these were good or positive coordination aspects of the policy dialogue they were involved in. These findings are in line with the current literature on policy dialogue, which regards a neutral facilitator, a broad mix of stakeholders and a clear goal as important features of a policy dialogue process [1, 6, 11, 12].

Another key finding of the study was that there lacked common understanding among the participants on the institution that led or coordinated the policy dialogue process. This can be seen as a weakness of the coordination process as Bokyo et al. [12] identify transparency as a key aspect of the policy dialogue process. Transparency in this case entails that participants understand who is leading and conducting the dialogue process. This is particularly important as it can help build the trust of participants on the dialogue process and its overall purpose.

Boyko et al. [12] also identify other aspects such as timeliness of a policy issue (to ensure its pertinence and appropriateness), availability of adequate resources, and equipping participants with the necessary resources and documents to engage in a dialogue, as important features of a policy dialogue. Inadequate financial resources, limited availability of documentation and inadequate time allocation for the dialogue, which were reported by the respondents in our study as the key constraints of the policy dialogue process, are bound to affect the success of a dialogue, according to Boyko et al. [12], who consider these aspects to be essential for a good policy dialogue process and which should be adequately planned for in the coordination of any policy dialogue.

While the factor of time was identified in terms of timeliness of a policy issue, our study also identified time as the amount of time allocated to the policy dialogue, as an important element for an effective policy dialogue process engendering quality and constructive exchanges. While no suggestions can rightfully be made as to what constitutes an adequate amount of time for dialogue as this may depend on the complexity of the issue being discussed, and the number and profile of stakeholders present; institutions coordinating a policy dialogue process can nonetheless conduct a quick survey among the stakeholders prior to the dialogue to assess what they believe could constitute an adequate amount of time for discussion and exchanges in the policy dialogue process they are to take part in.

Our findings also show that the coordination of activities (and the activities themselves) of the policy dialogue process effectively came to a halt as a result of the Ebola epidemic. These activities only resumed once concrete steps were taken by the WHO to re-animate the CCSS through hiring a health systems expert to help the CCSS continue its coordination task of implementing the policy dialogue process to revise and update Guinea’s national health development plan. This finding, in view of the other operational coordination challenges that respondents perceived during the policy dialogue, raises questions as to whether the CCSS had the required capacity (i.e., administrative and institutional support) right from the start, to effectively coordinate the policy dialogue process. It also raises questions about the institutional ownership of the policy dialogue process.

Yadav et al. [13] in describing the successful initiative of a national coalition for sustained optimal iodine intake in India, highlight the good organizational aspects of the national coalition which partly played a role in its success. These key organizational aspects include (i) having one partner agency assume leadership role; (ii) having a clear set of proposed duties or activities (terms of references) for the coalition; and (iii) an effective secretariat to support the work of the coalition (hosted within the lead agency) with a full time coordinator and part time secretarial staff, all, with clear terms of references and guided by senior experts with long term experience. The importance of these administrative and institutional tools in facilitating good coordination have also been documented in other fields of work, for example by Sunderwall et al. [14, 15] on aid coordination in Zambia and Bangladesh, who also highlight the instrumental role of using memoranda of understanding or terms of references that clearly outline the roles and responsibilities of partners or agencies that are part of the broader framework, committee or coalition. The importance of government ownership and leadership as a key component for effective coordination of dialogue processes has also been emphasized in the literature [16–18]. While these studies are not focused on policy dialogue processes per se, they do offer useful insights on key principles and determinants that facilitate good coordination which that can be used to improve the coordination of any policy dialogue process.

Some of the operational challenges identified by the respondents during the policy dialogue process (limited documentation, financial delays and insufficient time), could have emanated in part from the unavailability or limited capacity of the secretariat (and/or a full time coordinator) to support the coordination tasks of the CCSS. The fact that the respondents did not have clear or common knowledge as to which institution coordinated the process, may have been the result of unclear terms of references for the CCSS or maybe the lack of a clear memorandum of understanding among the organisations or stakeholders who were part of CCSS. Such a memoranda between the MoH, WHO, EU and other participating institutions clearly specifying which institution was the lead agency, would better support the coordination role of the policy dialogue process. In effect, the CCSS was created as a multi-stakeholders committee, meaning that actors with different institutional affiliations were part of this committee. The fact that the institutionalization of the CCSS at both national and subnational level had not yet been fully established prior to the Ebola outbreak, supports the idea that the CCSS may not have had the administrative and institutional support necessary to effectively coordinate the dialogue process. This might explain some of the operational coordination challenges that were faced during the policy dialogue process.

The findings of this study has implications for the leadership role of the Ministry of Health of Guinea as it continues on the process of policy dialogue to enhance the development of evidence-based policies and plans and as it moves forward with the health systems strengthening agenda. The findings of our study highlight the importance of understanding the the practical and operational underpinnings of coordination. There is a need for more research to be conducted in this light if we are to gain good understanding about how to concretely strengthen the coordination capacities of institutions or structures particularly for such processes in the context of LMICs.

### Strengths and limitations

Our study reflects results that refer to only one country which may limit the generalizability of our findings to other settings. We however believe that the fact that we interviewed senior and knowledgeable officers, who had been part of the policy dialogue process, provides real life experiences that further research can build on, as well as serve as a basis for strengthening policy dialogue process in low income countries.

## Conclusions

This study examined the coordination of the policy dialogue process in developing the key strategic, governance documents of the Guinea health sector from the perspective of actors involved in the policy dialogue process. The results suggest that there were both strengths and weaknesses in the coordination of the policy dialogue process and highlights the importance of ensuring that structures or committees that coordinate a dialogue process are given (or have) the relevant administrative and institutional support and leadership, to effectively coordinate a sustained dialogue process among a broad range of stakeholders. Time and financial resources must be availed to facilitate the coordination process, evidence must be generated to inform the dialogue and, supportive documentation shared with stakeholders in a timely manner to realise meaningful discussions. Further research in contexts where these parameters exist will provide more insights on the extent to which they are valid.

## Abbreviations

CCSS, Coordinating Committee of the Health Sector; CNRE, Cellule nationale de coordination de la maladie à virus Ebola; MoH, Ministry of Health; NHDP, national health development plan; WHO, World Health Organization
